# Effect of Environmental and Anthropic Conditions on the Development of *Solanum peruvianum*: A Case of the Coastal Lomas, Lima-Peru

**DOI:** 10.3390/plants13192683

**Published:** 2024-09-25

**Authors:** Vladimir Camel, July Quispe-Huañahue, Edwin Felix, Zulema Ninanya-Parra, Yngrid Mendoza, Sebastian Peralta-Yalta, Freddy Pillpa, Rita Cabello-Torres

**Affiliations:** 1 Grupo de Investigación en Ecofisiología Vegetal y Restauración de Ecosistemas Degradados, Escuela de Ingeniería Ambiental, Universidad Cesar Vallejo, Lima 15434, Peru; jquispehu01@ucvvirtual.edu.pe (J.Q.-H.); efelixhu@ucvvirtual.edu.pe (E.F.); zuganp@gmail.com (Z.N.-P.); ymendozahe29@ucvvirtual.edu.pe (Y.M.); jperaltaya@ucvvirtual.edu.pe (S.P.-Y.); fpillpaa@ucv.edu.pe (F.P.); rcabello@ucv.edu.pe (R.C.-T.); 2 Programa de Fisiología Vegetal y Cambio Climático, Asociación ANDINUS, Calle Miguel Grau 370, Sicaya 12500, Peru

**Keywords:** degraded soils, organic amendments, photosynthesis, restoration of degraded areas

## Abstract

Land degradation and the effects of climate change are increasing arid lands, accelerating desertification, and leading to the loss of ecosystem services worldwide. This research focused on evaluating how human impact and environmental factors affect the development of *Solanum peruvianum* in its natural habitat of coastal lomas. The study was carried out in the coastal lomas of Mangomarca-Peru, where phenotypic and ecological data on the plants were collected. Information was also gathered on human impacts on the nutritional characteristics of the soils. Then, five types of organic amendments were used to improve the physical and chemical characteristics of the degraded soil, and the development and photosynthetic activity of *S. peruvianum* were evaluated. As a result, under the study conditions, it was found that *S. peruvianum* was established approximately 33.74 cm from the rocks, in a range of 300 to 650 m asl. The maximum height of the plants was 90 cm, with a stem diameter at ground level of 2 cm. *S. peruvianum* produced fruits between January and July, with a seed germination rate of 36% in 25 days. On the other hand, the anthropogenic impact on the soil reduced 58% of organic material (OM), 71% of nitrogen, 40% of P_2_O_5_, and 13% of K_2_O and increased the concentration of magnesium oxide, calcium oxide, pH, and electric conductivity (EC). The organic amendments bokashi, compost, and biochar, when mixed with the degraded soil, increased the pH, OM, N, P, and EC; however, the plants died after 25 days. On the other hand, the application of the Premix5 substrate for 100 days favored the growth of 52.84 cm and 38.29 cm in the preserved soil and 23.21 cm in the black soil mixed with blond peat, and it should be noted that the substrates presented an acid pH and EC > 0.1. Regarding photosynthetic phenotyping, *S. peruvianum* plants grown in their natural habitat and in Premix5 showed a higher proton flux (vH+), linear electron flow (LEF), and maximum quantum yield (Fv’/Fm’). On the contrary, they showed a lower NPQt value than plants grown in preserved and black soil mixed with blond peat.

## 1. Introduction

Arid soils cover 41% of the Earth’s surface and have recently expanded due to anthropogenic impacts and global warming [1]. Likewise, the frequency of droughts, floods, fires, desertification, etc., is increasing [2,3], generating different types of biotic stress (pathogens and pests) and abiotic stress (water, thermal, saline, radiation, etc.) to plants, which accelerates the degradation, fragmentation, and loss of ecosystem services [4]. This situation represents a direct threat to the availability of fresh water, food security, and sanitary conditions of human society [5,6,7]. For this reason, the United Nations considers this decade (2021–2030) as the period for the restoration of ecosystems, and its objective is to recover environmental services and mitigate the effects of climate change for the benefit of humanity and other biodiversity [8].

In this sense, a peculiar ecosystem of the arid coast of Peru is addressed: coastal lomas, also known as fog oases [9] or meadows of vegetation in the middle of the desert [10]. The coastal lomas are located in one of the most arid regions in the world, distributed between 0 and 1300 m above sea level from northern Peru near Trujillo at the 7° S parallel (Peru) to Coquimbo (Chile) at 30° S [11]. They also appear discontinuously on nearby hillsides of the sea [12]. Currently, the coastal lomas in Peru cover approximately 2000 km^2^ [10]. However, five centuries ago, they occupied more than 15,000 km^2^ [10]. On the other hand, the annual vegetation of the coastal lomas develops during humid periods (between June and September) under the influence of fog [10,12]. It is also characterized by a high endemic diversity of flora (*Ismene amancaes*, *Atriplex rotundifolia, Heliotropium arborescens, Haageocereus acranthus, Vasconcellea candicans, Mimosa alvida,* etc.) and fauna (*Microlophus tigris, Platalina genovensium*, *Glossophaga soricina*, *Rhodopis vesper*, *Pseudasthenes cactorum*, etc.), which are vulnerable to prolonged droughts and anthropogenic impact [13], for example, the increase in urban areas, road opening, PM10 particulate material, and trampling by animals and humans. This study focused on the species *Solanum peruvianum,* a type of perennial wild tomato that grows in extremely arid habitats under relatively low evaporation demand [14]. *S. peruvianum* presents high genetic variability and physiological plasticity, which allows it to be tolerant to drought, high temperatures, and salinity [15,16,17]. Genetically, it is resistant to early blight, brown root rot, Septoria leaf spot, root-knot nematodes, bacterial canker, and bud necrosis disease. It also has genes for resistance to mosaic virus [18]. On the other hand, some physiological studies indicate that the main limiting factors for development are light, water, and nutrients [19,20]. At the photosynthetic level, fertilization in tomatoes helps to increase the chlorophyll content and the net photosynthetic rate and, therefore, generates greater production of NADPH, ATP, and carbohydrates [21,22]. However, under natural growth conditions, its ecology, photosynthetic physiology, hydraulics, and plant-microorganism interaction, among other relevant aspects, are still unknown. Then again, *S. peruvianum* is important in the ecosystem of coastal lomas since it contributes to soil conservation and the phytostabilization of heavy metals such as Pb, Cu, and Cd [23]. This represents a good option for recovering degraded soils in coastal lomas. These ecosystems are disturbed by the increase in urban boundaries, which remove the biological crust of the layer of organic matter (OM) and alter the soil’s physicochemical properties, increasing the loss of vegetation cover. Consequently, these changes negatively influence the capacity to retain water and nutrients [24,25,26].

It is important to note that soils store 30% of carbon in forests and more than 90% in grasslands and agricultural areas. Therefore, it is important to recover degraded soils to conserve bacterial biomass and optimize the biochemical cycles of nutrients [27,28]. It is necessary to develop fertilizers that improve the quality of soils, considering that their origin, age, management, and humidity, among other factors, affect the ecosystem [21]. According to some studies, the application of different organic fertilizers, such as compost, bokashi, and biochar, is an alternative for combating soil degradation [29,30,31]. Recycled materials are used to produce organic fertilizers, reducing environmental pollution. Additionally, these amendments maintain the balance in the proportion of carbon and nitrogen, provide organic matter, allow greater microbial activity, improve carbon sequestration, increase resilience to extreme weather events (drought and substantial rainfall), and reduce the emission of greenhouse gases (CO_2_, CH_4_, and N_2_O) compared with the use of synthetic fertilizers [32]. Finally, the restoration of coastal lomas is relevant for the city of Lima, the capital of Peru, since it generates the largest amount of carbon dioxide (2.71e^+7^Tn per year) in the country [33], and its average green areas per individual are 3 m^2^ per capita [34], which is below others reported, such as the capital of the Netherlands (17.62 m^2^ per capita) [35], cities in the interior of the Mongolian plateau (>20 m^2^ per capita) [36], and the city of Tczew in northern Poland (70.6 m^2^ per capita) [37], among others.

This research evaluated how human impact and environmental factors affect the development of *S. peruvianum* in its natural habitat of coastal lomas, as well as the effect of applying organic amendments on the development of *S. peruvianum* and the physical-chemical properties of the degraded soil. We tested the following hypotheses: (i) Environmental factors and human disturbances affect the population structure of *S. peruvianum*, with higher plant sizes expected in lower slopes and more preserved zones. Furthermore, (ii) the fertilization of degraded soils improves nutritional properties, helping the establishment, development, and optimization of the photosynthetic capacity of *S. peruvianum* plants.

## 2. Results

### 2.1. Population Structure of S. peruvianum

**Figure 1:**
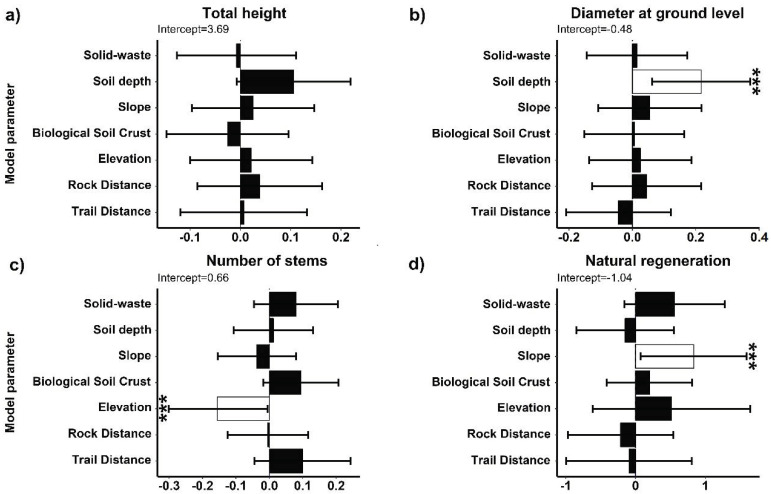
Average coefficients of the model that influence the parameters of the structure of *Solanum peruvianum*: (**a**) total height, (**b**) diameter ground level, (**c**) number of stems, and (**d**) natural regeneration. Error bars represent 95% confidence intervals. Black boxes with asterisks indicate significant effects on structure parameters (*p* < 0.05).

The density of *S. peruvianum* individuals in the Mangomarca Lomas was approximately 150 plants. The species was distributed along an elevation gradient between 300 and 650 m asl. The ground-level diameter increased as soil depth increased (Figure 1a). Elevation hurt the number of branches (Figure 1b,c), while slope positively affected the amount of plant regeneration (Figure 1d,e). No anthropogenic disturbance variable (roads, solid waste) or microsite characteristic (distance from rock and biological crust) showed any effect on the phenotypic characteristics of *S. peruvianum* (basal area, diameter, maximum diameter, total height). However, *S. peruvianum* establishes near rocks at an average distance of 33.74 cm, on soil with an average slope of 43.54%, and at a depth of 6.5 cm.

### 2.2. Differences in Physicochemical Properties between Preserved and Degraded Soils in Coastal Lomas

**Figure 2:**
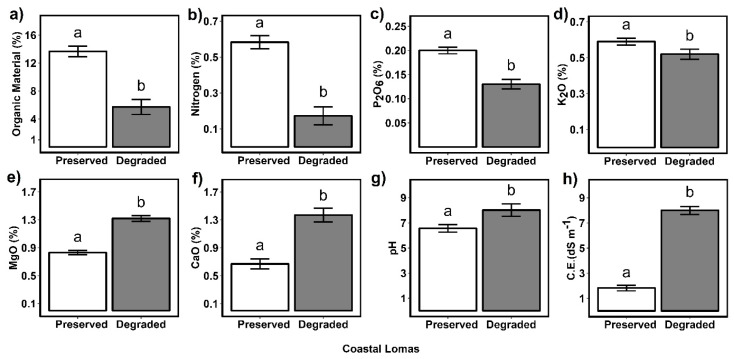
Comparisons of soil characteristics in preserved and degraded coastal Lomas: (**a**) organic material (%), (**b**) nitrogen (%), (**c**) diphosphorus oxide (P_2_O_5_), (**d**) potassium oxide (K_2_O %), (**e**) magnesium oxide (MgO %), (**f**) calcium oxide, known as quicklime (CaO %), (**g**) pH, and (**h**) electric conductivity (C.E. dS m^−1^). Different letters are significantly different at *p* ≤ 0.05, as per Tukey’s test after GLMM. Error bars represent the 95% confidence intervals.

The results showed negative changes in the chemical structure of the soil in the Mangomarca Lomas due to human impact. The conserved soil presents an average of 13.67% ± 0.49 of organic matter, while the degraded soil contains 5.71% ± 0.69, showing a decrease of 58%. Likewise, the percentage of nitrogen was reduced by almost 71%, P_2_O_5_ decreased by 40%, and K_2_O decreased by 13% (Figure 2d). In contrast, degraded soils showed a greater increase in the concentration of magnesium oxide (MgO), calcium oxide (CaO), pH, and electrical conductivity. Regarding pH, degraded soils were found to be alkaline, with an average value of 8.02 ± 0.05, while preserved soils were slightly acidic, with an average value of 6.56 ± 0.03. Electrical conductivity was also affected by anthropogenic impact. The degraded soil presented an average of 8 ± 0.31 dS m^−1^, while in conserved soil, it was 1.82 ± 0.22 dS m^−1^. These reports show that in degraded soils, as the pH increases, the EC values also increase (Figure 2g,h). These changes affect nutrient exchange processes from the soil to the plant. These results are consistent since as calcium oxide values increase, pH levels also increase. Therefore, these changes affect the survival rate of plants commonly adapted to slightly acidic soils.

### 2.3. Application of Organic Fertilizers on the Chemical and Biological Properties of the Degraded Soil of the Mangomarca Lomas

The effect of soil degradation on lomas due to anthropogenic activities has been evidenced by modifying their physical-chemical and biological properties. Therefore, in this research, we sought to improve nutritional conditions by applying different organic fertilizers and substrates (Table 1) for the subsequent planting of *S. peruvianum* plants. The chemical results show that all treatments considerably increased the values of organic matter, nitrogen, K_2_O, and P_2_O_5_, bringing them close to the values of the conserved soil. There were no changes in magnesium oxide (MgO) compared with those in degraded soil (Table 1). On the contrary, the organic fertilizers used increased the pH and electrical conductivity levels, exceeding the averages of 7.76 ± 0.31 and 8.02 ± 0.4 dS m^−1^, respectively. Contrary to this, using Premix5 and black soil mixed with blond peat showed a considerable reduction in pH and electrical conductivity.

**Figure 3:**
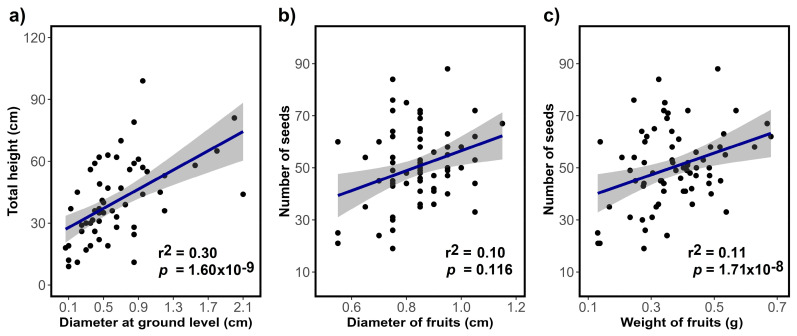
Morphological evaluation of *Solanum peruvianum*. (**a**) Correlation between diameter at ground level and total height; (**b**) Relationship between fruit diameter and number of seeds. (**c**) Correlation between fruit weight and number of seeds. The grey band indicates the 95% confidence limit.

However, the *S. peruvianum* species was used as an indicator to evaluate the efficiency of the substrates mixed with the degraded soils. Under natural growth conditions, *S. peruvianum* presented a significant correlation between the ground-level diameter and the total height (r^2^ = 0.30, *p*-value *=* 1.60 × 10^−9^, Figure 3a). Individuals with a maximum size of 90 cm and a diameter of 2.00 cm were recorded.

On the other hand, it is important to note that the presence of *S. peruvianum* fruits was recorded between January and July. From a group of 3567 seeds, 1284 seeds germinated (36% germination) in 25 days. The analysis regarding the number of seeds and the fruit diameter variables (Figure 3) produced a low coefficient of determination (r^2^ = 0.10; *p* = 0.116). Likewise, the weight of the fruits and the number of seeds produced a significantly low coefficient of determination (r^2^ = 0.11; *p* = 1.71 × 10^−8^).

**Figure 4:**
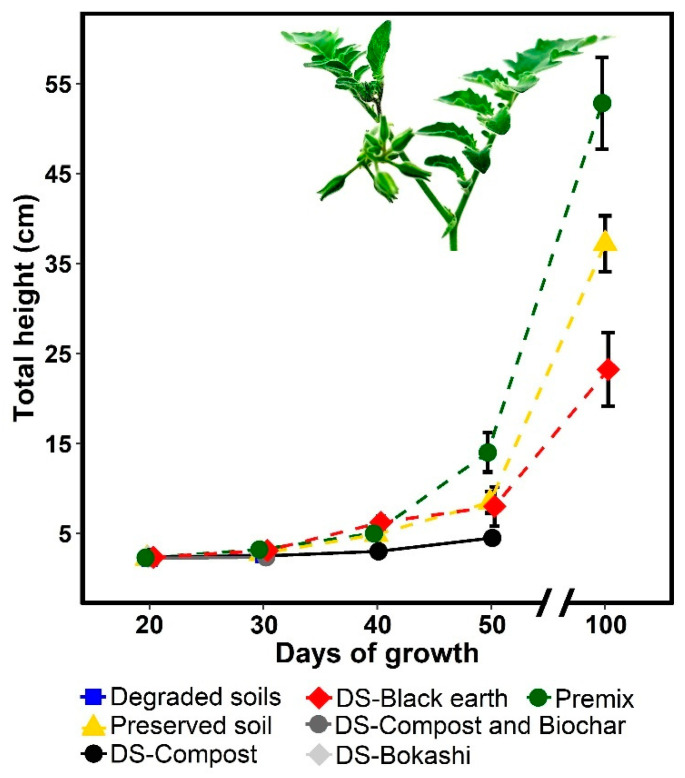
Statistical differences in the growth of *S. peruvianum* under different organic substrates.

According to the experimental design, germinated *S. peruvianum* plants were placed in trays for one month, then subjected to the treatments formulated in this research (Table 1) and monitored for 100 days. The results showed growth of 52.84 cm in the Premix5 substrate, 38.29 cm in preserved soil, and 23.21 cm in degraded soil mixed with black soil and blond peat (Figure 4); it is important to note that the three substrates corresponded to acidic soils with low electrical conductivities. On the contrary, in all bare soils, the plants died.

**Figure 5:**
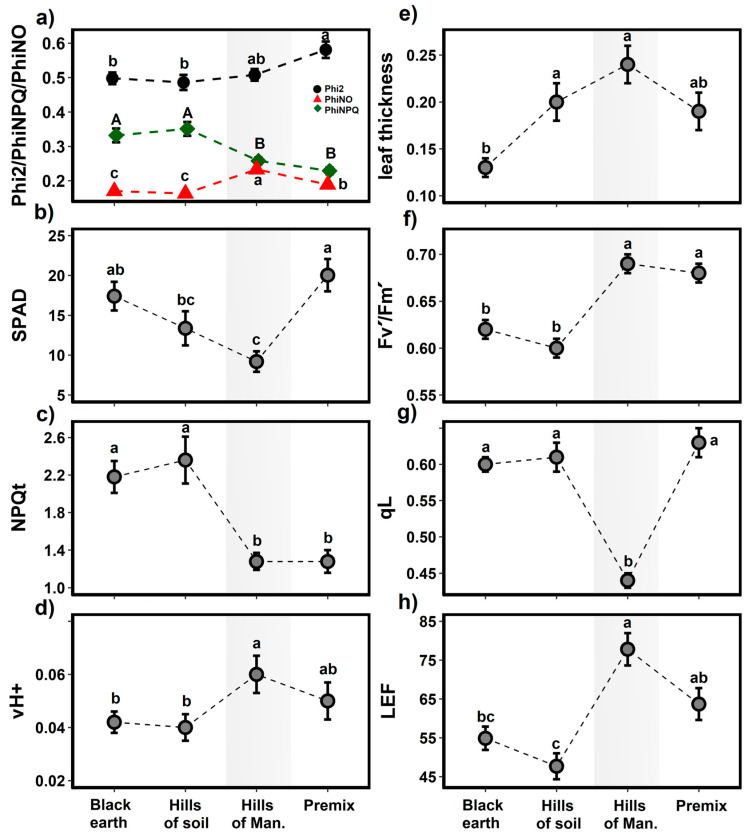
Photosynthetic performance of *S. peruvianum*: (**a**) PSII; (**b**) relative chlorophyll; (**c**) total non-photochemical quenching corrected for Fm (NPQt); (**d**) proton conductivity of the chloroplast ATP synthase (vH+); (**e**) leaf thickness; (**f**) maximum quantum efficiency (Fv’/Fm’); (**g**) fraction of photosystem II open center (qL); (**h**) linear electron flow (LEF). Different letters significantly differ at *p* ≤ 0.05 with Tukey’s test after GLMM. The grey band indicates photosynthetic measurements in the natural habitat of *S. peruvianum*, in the Mangomarca Lomas.

The results of the photosynthetic phenotyping of *S. peruvianum* plants grown naturally in the Mangomarca Lomas showed greater leaf width, proton flux through the thylakoid membrane (vH+), linear electron flux (LEF), and maximum quantum yield (Fv’/Fm’) despite presenting a lower concentration of chlorophyll and fraction of open PSII centers (qL) (Figure 5). It should be noted that the plants in the Mangomarca Lomas are not subject to irrigation, contrary to the laboratory’s controlled experimental conditions. Despite the arid climatic conditions of the Mangomarca Lomas, the *S. peruvianum* species presented a lower NPQt value than the plants grown in the different substrates applied in the laboratory (black soil and blond peat and in the soils of the Lomas). Meanwhile, the results also indicate that in the Premix5 substrate, the *S. peruvianum* plants achieve greater development and photosynthetic activity (Figure 5). However, the soil of the Lomas, rich in nutrients, did not differ in photosynthetic conditions from the substrate of blond peat and black soil.

## 3. Discussion

Although the ecosystem of the Mangomarca Lomas is considered fragile, it is being impacted by human activities throughout its entire extension (roads, solid waste, introduction of exotic species, etc.). However, microsite environmental variables such as soil depth, slope, and elevation had the greatest influence on the growth and development of *S. peruvianum* (Figure 1). Furthermore, the results indicate that plants with a larger stem are established in spaces with greater effective soil depth. Virgin soils have a high accumulation of OM [24]. Specifically, the Mangomarca Lomas are seasonal ecosystems; at the end of the wet season, the annual plants die [12], and their biomass becomes part of the nutrient mineralization process [38]. According to Sharanappa [39], OM improves soil structure by increasing water retention, supplies essential nutrients, introduces microorganisms responsible for nutrient mineralization processes, and improves disease resistance. These factors positively influence the proper development of roots and, consequently, the increased growth of *S. peruvianum* plants. The results also show that the slope has a positive influence because it generates a greater presence of natural regeneration. The steepest slopes in the Mangomarca Lomas are associated with rocks, which suggests a significant potential for providing soluble fertilizers [40] and establishing a plant regeneration process.

In a study led by Ángel et al. [41], it is mentioned that rocks benefit the survival of *Bursera* spp. compared with those established under a nurse plant or in open areas. According to the present study, all *S. peruvianum* plants are established near rocks between a distance of 0 and 100 cm. Rocks regulate temperature and generate favorable plant habitats [42]. In the summer, they produce shade, reducing direct solar radiation and soil moisture loss [43]. Due to the presence of winter fog on the coastal lomas [10], the rocks intercept the mist, creating a microclimate with greater relative humidity in the soil, thus aiding seed germination. Likewise, rocks provide essential minerals to plants [44] and act as physical obstacles that slow down soil erosion, reduce wind speed, and trap seeds [45], favoring germination, growth, and development in *S. peruvianum* individuals. The research results, therefore, reinforce the importance of rocks as suppliers of nutrients and minerals, as well as an important habitat in the Mangomarca Lomas.

Although approximately 34 regenerations of *S. peruvianum* were reported in the inventory, it is important to note that under laboratory conditions, only 36% of the seeds germinated within 25 days. In comparison with other studies, Campbell et al. [46] indicated that *S. mauritianum* seeds germinate at 77.03% in the presence of light and within a temperature range of 15–30 °C, while only 1% germinate under conditions of 20 °C and in darkness.

On the other hand, Ranil et al. proposed a germination protocol for *S. torvum* seeds considering soaking, gibberellic acid (GA3), potassium nitrate (KNO_3_), cold stratification (4° to 7 °C), thermal shock (37 °C), and light irradiation during germination of 16 h of light (25 °C)/8 h of darkness (18º C). This protocol reached 100% germination at six days; in contrast to seeds that did not receive treatments and did not receive illumination, they did not show any germination at 15 days. On the other hand, this research shows that anthropogenic impact directly affects soil properties (OM %, nitrogen %, P_2_O_5_, K_2_O, MgO, CaO, pH, and EC). According to previous studies, the soils of the coastal lomas have an acidic pH and high values of OM, phosphate, and potassium. In the hill of Mollendo, the organic carbon content was around 6.50% and had a pH of 4.7 [47], while in the hill of Atiquipa, an approximate 3.18% of OM and a pH of 4.9 was reported within the remaining forests of *Caesalpinia spinosa* [48]. Nevertheless, high pH values (>7.5) were found in soils from the lomas of Villa María del Triunfo, while low pH values (<5) were found in soils from the Lachay lomas [49]. In contrast, the soils from the Mangomarca Lomas contain an average of 13.67% OM, have an EC of 1.08 ± 0.04 dS m^−1^, and have a pH of 6.56. The Mangomarca Lomas’ pH facilitates most plants’ biochemical processes since the favorable pH for most crops is 6 to 7 [39].

The results of the present research are in agreement with the studies by Kakeh et al. [50] conducted in Alagol grassland in Sahra Turkmenistan, where pH, EC, Ca, and Mg concentrations were lower in biocrusted soils than in degraded soils, but nitrogen and phosphorus were higher in biocrusted soils. Furthermore, biocrusts help in salinity reduction [51,52] and improve water infiltration and soil nutrient enrichment [50].

On the other hand, the current results show that the impacted soils reduced their OM concentration to 5.71%. The OM of the soil contains more than three times the carbon of the atmosphere [53], which implies that urban growth has considerably altered the CO_2_ sinks in the Mangomarca Lomas. Despite the impacts of man, 5.71% of OM is higher than that of the other coastal lomas reported. However, our results indicate that under basic pH conditions, high EC, CaO, and MgO values lead to the death of *S. peruvianum* plants. According to [39], saline soils have an EC greater than four and a pH greater than 8.2; high concentrations of some cations cause a reduction in the absorption of other nutrients (N, P, K) that may be essential for plant growth. Blossom-end rot is associated with calcium deficiency, although stress at high concentrations of calcium salts can also cause blossom-end rot [54]. Both magnesium deficiency and excess can reduce leaf area [55]. The present study also reports that degraded soils had lower nitrogen, P_2_O_5_, and K_2_O concentrations. Nitrogen, phosphorus, and potassium are the macronutrients necessary for plant growth and development [56]. Likewise, nitrogen deficiency causes leaf chlorosis and etiolation. Phosphorus deficiency results in retarded shoot growth and branching, dark green to blue leaf discoloration, weaker and thinner stems, and reduced fruit quantity and quality. Potassium deficiency causes yellowing of leaf margins and necrotic growth of older leaves [54].

On the other hand, photosynthetic parameters were measured after 100 days of plant growth. The results indicated that *S. peruvianum* plants that grew naturally on coastal lomas had a higher proton and electron flow capacity, a higher maximum quantum yield, and a low level of NPQt and % relative chlorophyll. Furthermore, the plants were flowering and fruiting under climatic conditions of 32 degrees maximum temperature, 1500 PAR, and 5% humidity. However, microorganisms in the rhizosphere of *S. peruvianum* could play an important role in tolerance to water and salt stress. According to some studies, host plants and their associated microbes co-evolve with each other, constituting a “holobiont” that adapts in response to ongoing environmental changes [57,58]. Some studies showed that a single strain of M. alpine significantly improves plant growth under drought stress by optimizing carbohydrate and nitrogen biosynthesis. Thus, by improving carbon and nitrogen acquisition, plants strengthen their capacity and flexibility to cope with various environmental stresses (de Vries, 2020). Organic amendments such as biochar, bokashi, and compost were able to increase the percentages of OM, nitrogen, P_2_O_5_, and K_2_O, thus being beneficial for plant nutrition; however, they showed a liming effect and EC equal to or greater than the degraded soil, which is characteristic of saline soils and harmful to the growth of *S. peruvianum*. In this regard, Killi et al. [31] reported that compost from olive solid residues maintained the pH and raised 10% of the EC in sandy soils to cultivate *S. lycopersicum*. Likewise, Baliran et al. [59] prepared bokashi from cow dung, wheat stover, bran, and other components, reporting a pH of 7.5 and an EC of 4.9. Additionally, Ibrahim et al. [60] reported that the use of biochar made from mango, Casuarina, and Salix increased the pH and EC values of the soil; however, the pH and EC can be reduced when the biochar is prepared at temperatures below 400 °C [61]. On the other hand, saline soils are only reclaimed by the leaching of salts through abundant water availability, good drainage, and the addition of fertilizers (N, P, and K) with low salt index [39].

## 4. Materials and Methods

### 4.1. Study Area

The study was carried out in the Mangomarca Lomas, located in the District of San Juan de Lurigancho, Lima Region (12°20′25″ S, 75°45′50″ W), Peru. It is a fragile ecosystem that extends along an altitudinal gradient from 250 to 850 m above sea level (m asl). It is home to approximately 26 families of vascular plants with 44 genera and 51 species, of which eight are endemic. During dry periods, the species *Haageocereus acanthus*, *Vasconcella candicans*, *Solanum peruvianum*, *Nicotiana paniculata*, *Trixis cacalioides*, *Mimosa albida*, and *Atriplex rotundifolia* predominate. During the humid period, species such as *Ismene amancaes*, *Solanum montanum*, *Solanum tuberosum*, *Sicyos baderoa*, *Salvia paposana*, etc. emerge. Likewise, Cabello-Torres et al. [62] warned of a reduction in vegetation cover not only due to climatic issues but also due to land trafficking and indiscriminate invasions due to anthropogenic pressure, which causes a risk to its conservation.

The inventory was conducted between August and September 2023. Three 100-m linear transects were established along the trails at elevations between 200 and 500 m asl.

Visual plant encounter censuses were developed to avoid damage to the ecosystem and generate essential information on the Mangomarca Lomas. The total area evaluated was 24 ha. In each transect, plant data were recorded, including diameter at ground level (DGL), total height (TH), number of branches, altitude, and presence/absence of fruits and flowers. Height estimates were calculated using a millimeter ruler. The distance to rock, distance to trail, presence/absence of solid waste, presence/absence of biological crust, and soil depth were also evaluated. The altitude values of each individual were estimated using a Garmin 76SX.

### 4.2. Soil Sample Collection

**Figure 6:**
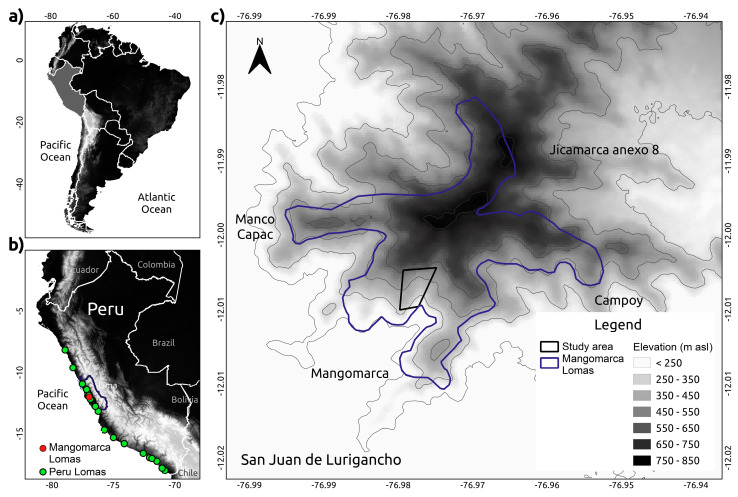
*S. peruvianum* plants and location of coastal lomas ecosystems in Peru. (**a**) Location of Peru in South America. (**b**) On the map of Peru, green dots indicate the position of other coastal lomas, and red dot indicates the position of the Mangomarca Lomas. (**c**) Map of the coastal lomas of Mangomarca. The polygon indicates the region where the *S. peruvianum* plants are distributed.

For the collection of soil samples, six areas of 100 m^2^ were identified through an altitudinal gradient, three areas were identified to collect samples of preserved soil (12°00′08.83″ S, 76°58′44.72″ W; 11°59′57.17″ S, 76°58′20.46″ W and 11°59′48.99″ S, 76°58′18.42″ W), and three were identified to collect samples of degraded soil (12°00′14.74″ S, 76°59′10.12″ W; 12°00′05.89″ S, 76°58′53.43″ W and (12°00′14.74″S, 76°59′10.12″ W). The areas of anthropic impact were characterized by being close to roads and housing construction, in addition to having the presence of dead plants of *Haageocereus acranthus* (Figure 6) since it is a species of cactus present in the preserved lomas and is an indicator of the state of conservation of the coastal lomas. On the other hand, ten soil samples of ½ kg per area were collected at a depth of 15 cm and deposited in a plastic container. Then, the samples in the container were divided into four equal parts and discarded into two portions. In comparison, the remaining two portions were remixed and divided again into four equal parts, and the two opposite parts were discarded. This procedure continued until four parts of ½ kg of preserved and degraded soil remained. Subsequently, the samples were dried and sieved (2 mm), and finally, the physical and chemical characteristics (OM %; nitrogen %; phosphorus pentoxide, P_2_O_5_ %; potassium oxide, K_2_O %; magnesium oxide, MgO %; calcium oxide, known as quicklime, CaO %; pH; and electric conductivity, EC, dS m^−1^) were analyzed with three replicates for each variable in the soil laboratory of the Universidad Nacional Agraria La Molina.

### 4.3. Treatment of Degraded Soils in the Development of S. peruvianum Plants

The experimental design comprised seven treatments, and 50 plants were established for each treatment as repetitions. In T1, the reference soil and perlite were mixed in a 2:1 proportion; T2 was a mixture of degraded soil and perlite (2:1); for T3, the degraded soil was mixed with bokashi and perlite in a 2:2:1 proportion; T4 presented a proportion of degraded soil, compost, and perlite (2:2:1); T5 presented a concentration of degraded soil, compost, *Schinus molle* biochar, and perlite in a 2:2:1:1 proportion; T6 mixed samples of black soil, blond peat, degraded soil, and perlite (2:2:1:1); and, finally, for T7, the substrate Premix5 was used.

The samples’ chemical analysis (OM %, nitrogen %, P_2_O_5_, K_2_O %, MgO %, CaO %, pH, and EC dS m^−1^) was carried out in the Soil Laboratory of the National Agrarian University La Molina. 

The evaluation of the development of the *S. peruvianum* plants in the Mangomarca Lomas included identifying 76 plants in good phytosanitary conditions. A fruit was collected for each plant, considering its maturity. Then, two fruit diameter and weight measurements were taken using an analytical balance. Next, the seeds were extracted and counted. Then, the seeds were washed with distilled water and placed in germination chambers at 25 °C. Germination was evaluated for 25 days. Finally, the seeds were transplanted into tomato seedbeds measuring 5 cm × 5 cm using Premix5 for tomatoes as a substrate. They were then transplanted to different treatments and controls.

### 4.4. Growth Evaluation and Photosynthetic Phenotyping of S. peruvianum

The total height growth was monitored for 100 days. Photosynthetic parameters were measured between 11 a.m. and 2 p.m., first identifying the most developed leaf.

All measurements were performed using the MultispeQ equipment (PhotosynQ is a Michigan State University (U.S.) Innovation Center startup that allows researchers, educators, farmers, and others to collect and discuss photosynthesis data, https://www.photosynq.com). These include the Quantum yield of Photosystem II (Phi2), the yield of non-photochemical quenching (PhiNPQ), the yield of non-regulated energy dissipation (PhiNO), linear electron flow (LEF), steady-state rate of proton flux flow through the chloroplast ATP synthase (gH+), proton conductivity of the chloroplast ATP synthase (vH+), total magnitude of ECS (electrochromic shift) decay during a light–dark transition (ECSt_tAU), and relative chlorophyll content (SPAD) (Table 1). The climatic variables (temperature, humidity, and radiation) were recorded using the MultispeQ equipment.

### 4.5. Statistical Analyses

To assess how chemical properties vary between degraded and preserved soil, we fitted linear models (LMs). Models were run using a Gaussian error distribution with an identity link function (normality was tested and confirmed using the Shapiro–Wilk test). Tukey post hoc tests assessed differences between chemical variables across treatments used to improve degraded soil. Then, we used mixed effects models to compare differences between photosynthetic parameters (PSII, LEF, qL, SPAD, NPQt, vH+) of *S. peruvianum* plants across treatments. To account for potential non-independence, we included the plant as a random factor.

Mixed effects models were used to examine microsite conditions (bare soil, rock cover, grass cover, slope, elevation) affecting the development of *S. peruvianum* (basal area, frequency of individuals, diameter, height, and maximum height). Given the possible lack of independence among sampled individuals, transects were considered random factors. The homogeneity of variance was verified using residual plots. The effects of anthropogenic and environmental impact and the importance of each variable were analyzed from a multiple model inference approach based on the Akaike information criterion (AIC) with a correction for small sample size (AICc). The models were ranked from best to worst, and the set of models with ΔAICc ≤ 4 was considered. The Shapiro–Wilk statistical test verified the data’s normality. All analyses were performed using the R-project program version 4.4.1.

## 5. Conclusions

In Mangomarca coastal Lomas, there are approximately 150 *S. peruvianum* plants, distributed between 300 and 650 m above sea level, established near the rocks at an average distance of 33.74 cm, with an average slope of 43.54% and at an average soil depth of 6.5 cm. *S. peruvianum* presents a significant correlation between the stem diameter at ground level and the total height, with individuals reported with a maximum size of 90 cm and a diameter of 2.00 cm. Likewise, it presents fruits between January and July; its germination rate was 36% in 25 days. The plants with the largest stem diameter were found in the soils with greater depth, while the slope positively affected the amount of plant regeneration. Likewise, the results show that human impact reduced OM by 58%, nitrogen by 71%, P_2_O_5_ by 40%, and K_2_O by 13%. On the contrary, degraded soils showed a more significant increase in the concentration of magnesium oxide (MgO), calcium oxide (CaO), pH, and EC. These changes affect the nutrient exchange processes from the soil to the plant. Likewise, organic amendments were applied to improve soil conditions. However, the used fertilizers (bokashi, compost, biochar) increased the pH and electrical conductivity levels, exceeding the averages of 7.76 ± 0.31 and 8.02 ± 0.4 dS m^−1^, respectively. On the other hand, the use of Premix5 and black soil mixed with blond peat showed a considerable reduction in pH and electrical conductivity. However, *S. peruvianum* plants grew 52.84 cm in the Premix5 substrate, 38.29 cm in conserved soil, and 23.21 cm in degraded soil mixed with black soil and blond peat. In terms of photosynthetic phenotyping, the *S. peruvianum* plants that develop naturally in the Mangomarca Lomas have a greater leaf width, proton flux through the thylakoid membrane (vH+), linear electron flow (LEF), and maximum quantum yield (Fv’/Fm’). This situation develops despite presenting a lower concentration of chlorophyll and a fraction of open PSII centers (qL). It should be noted that the Lomas plants were not irrigated, contrary to the other experiments. Despite growing in arid climatic conditions, they had a lower NPQt value than plants growing in black soil and blond peat. On the other hand, the results also indicate that in the Premix5 substrate, *S. peruvianum* plants achieve more significant development and photosynthetic activity. However, the Lomas soil, which is rich in nutrients, did not differ in the photosynthetic conditions of the blond peat and black soil substrate.

## Figures and Tables

**Table 1:** Average nutrient, pH, EC, and organic matter values of the analyzed treatments. Different letters significantly differ at *p* ≤ 0.05 with Tukey’s test after GLMM (n = 3 replicates for each treatment). DS = Degraded soil; CS = Conserved soil; DSB = Degraded soil plus Bokashi; DSC = Degraded soil plus compost; DSC-B = Degraded soil plus compost and biochar; DSPBS = Degraded soil plus blond peat and black soil; Pre = Premix5.

Treatment	OM	Nitrogen	P_2_O_5_	K_2_O	CaO	MgO	EC	pH
**CS**	13.67 ^c^ ± 0.49	0.58 ^d^ ± 0.03	0.2 ^f^ ± 0.005	0.60 ^d^ ± 0.4	0.67 ^d^ ± 0.06	0.8 ^b^ ± 0.03	2.08 ^b^ ±0.27	6.56 ^e^ ± 0.3
**Pre**	50.82 ^d^ ± 0.69	2.25 ^e^ ± 0.04	0.05 ^g^ ± 0.007	0.18 ^e^ ± 0.03	0.76 ^d^ ± 0.09	1.06 ^c^ ± 0.05	0.90 ^c^ ± 0.19	5.44 ^f^ ± 0.4
**DS**	5.71 ^b^ ± 0.69	0.17 ^c^ ± 0.04	0.12 ^e^ ± 0.007	0.52 ^d^ ± 0.04	1.37 ^c^ ± 0.09	1.32 ^a^ ± 0.05	7.99 ^a^ ± 0.27	8.02 ^d^ ± 0.4
**DSB**	12.91 ^c^ ± 0.69	0.75 ^b^ ± 0.04	0.43 ^d^ ± 0.007	1.97 ^c^ ± 0.04	1.87 ^b^ ± 0.09	1.28 ^a^ ± 0.05	14.47 ^a^ ± 0.27	9.07 ^c^ ± 0.4
**DSC**	12.81 ^c^ ± 0.69	0.63 ^d^ ± 0.04	0.23 ^c^ ± 0.007	0.88 ^b^ ± 0.04	1.88 ^b^ ± 0.09	1.20 ^a^ ± 0.05	7.76 ^a^ ± 0.27	8.17 ^b^ ± 0.4
**DSC-B**	14.01 ^c^ ± 0.69	0.69 ^bd^ ± 0.04	0.26 ^b^ ± 0.007	1.20 ^a^ ± 0.04	2.15 ^a^ ± 0.09	1.28 ^a^ ± 0.05	8.29 ^a^ ± 0.27	8.52 ^a^ ± 0.4
**DSPBS**	72.28 ^a^ ± 0.69	0.91 ^a^ ± 0.04	0.16 ^a^ ± 0.007	0.54 ^d^ ± 0.04	1.57 ^c^ ± 0.09	1.03 ^c^ ± 0.05	0.91 ^c^ ± 0.27	5.53 ^f^ ± 0.4

## Data Availability

Data are contained within the article.
